# Short-Term Chronic Toxicity of Copper to *Hyalella azteca*: Contrast in Terms of Equilibrating Diet, Diet Type, and Organic Matter Source

**DOI:** 10.3390/toxics12080608

**Published:** 2024-08-20

**Authors:** Nafis Fuad, Rebecca Williams, Timothy M. Vadas

**Affiliations:** 1 Department of Civil and Environmental Engineering, College of Engineering, University of Connecticut, Storrs-Mansfield, CT 06269, USA; nafis.fuad@uconn.edu; 2 Department of Civil, Construction and Environmental Engineering, College of Engineering, North Carolina State University, Raleigh, NC 27695, USA; rlwill26@ncsu.edu

**Keywords:** heavy metals, toxicity, trophic transfer, *H. azteca*, dietary source, organic matter

## Abstract

The most up-to-date regulatory guidelines for establishing acute and chronic numeric limits for copper in freshwaters are based on a biotic ligand model for various species, but the model for Cu lacks data on dietary uptake. In addition, some common macroinvertebrate toxicity assay parameters are less representative of the ecosystem. We investigated the effects of diet and its type in the experimental setup and as an exposure pathway to an established amphipod (crustacean) *Hyalella azteca* (*H. azteca*) for Cu toxicity assays. We also investigated another overlooked aspect, the organic matter (OM) source. Our experiments compared the toxicity of pre-equilibrated and unequilibrated natural diets and a laboratory-favored diet in effluent and stormwater sources of organic matter adjusted to standard water characteristics. The experiments indicated a more toxic effect of the pre-equilibrated diet and natural dietary sources, and less toxic effects in the presence of effluent OM compared with stormwater OM, shifting LC50 or EC20 values by as much as 67% compared with the controls. The use of a pre-equilibrated natural diet in toxicity assays provides the advantage of producing toxicity data more representative of field conditions. Considering organic matter type, especially in dietary exposures, will better predict toxicity, accounting for copper complexation with OM from different sources and partitioning to the food supply. Adapting these ecologically relevant parameters in whole effluent toxicity testing or other assays will also provide safer regulatory oversite of discharges to surface waters.

## 1. Introduction

Copper is abundant in our environment, including surface waters, typically at natural levels in the single digits up to 10’s of μg L^−1^, or at increased levels due to anthropogenic activities (10 s of μg L^−1^) or mining activities (up to thousands of μg L^−1^) [1,2,3,4,5,6]. Copper is both essential at low levels [7] and toxic at slightly elevated concentrations to various organisms, including freshwater macroinvertebrates.

The bioavailability of Cu to organisms is controlled through aqueous speciation, exposure pathways, and the lifecycle of the organism. The most bioavailable species of aqueous copper is Cu^2+^ ions. However, aqueous Cu speciation is controlled by its strong affinity to ligands, especially in natural organic matter (NOM) [8,9,10]. The common exposure pathways are either aqueous or dietary [11], but the composition of the diet may be altered in the presence of aqueous Cu speciation through either adsorption sites that partition free copper from the aqueous phase or biotic uptake into the diet [12,13,14]. While all stream organisms experience aqueous exposures, macroinvertebrates can accumulate contaminants disproportionately from their diets compared with fish and small invertebrates, the two extremes of the trophic levels that are usually used in toxicity assays [11]. Currently, the U.S. Environmental Protection Agency (EPA) has established water quality criteria (WQC) for Cu based on toxicity assays conducted on one amphipod, one snail, and three Cladocerans using aqueous exposures with different chemical characteristics.

The established WQC for Cu used data from approximately 350 tests to derive normalized toxicity endpoint values (LC50 or EC50) for organisms including 15 species of invertebrates, 22 species of fish, and one amphibian species, representing 27 different genera. Nine of the ten most sensitive genera were invertebrates, and, in general, invertebrates were more sensitive than fish. Genus mean acute values (GMAVs) with cumulative probabilities closest to the fifth percentile toxicity value for all the tested genera were used to calculate a final acute value (FAV) for the WQC. However, this approach is purely focused on aqueous exposure and suffers from some ecologically relevant differences, such as organisms from different feeding classes, the effects of natural diet on aqueous exposure, and exposure via diet. These limitations likely lead to an underestimation of the toxic nature of Cu in surface waters under various contexts.

The current USEPA-published biotic ligand based Cu criteria [15] overlooks some important aspects of copper toxicity to aquatic macroinvertebrates. The minimum requirement is to use toxicity data for eight families of organisms, but this requirement has the risk of under-representing organisms from various trophic levels. In North America, freshwater invertebrate species outnumber freshwater fish species by eight to one and freshwater insect species by seven to one. Freshwater macroinvertebrates are an integral part of the other regulatory program, biological criteria, or biomonitoring. This disconnect between the two regulatory programs can have implications for the protectiveness of the WQC. For example, biomonitoring programs have shown that waters impaired with mountain-top coal mining waste do not support mayflies, while daphnids used in laboratory-based whole effluent tests showed those waters to be protective of aquatic life [16]. The different life stages of macroinvertebrates, their dietary behaviors, and the various environmental conditions supporting their whole lifecycles warrant careful consideration in toxicity testing in order to protect these organisms from contaminants at different life stages. For example, mayflies *N. triangulifer* consume a significant amount of their diet during the larval stage; however, they refrain from consuming at all during the last seven days before emerging. Furthermore, these organisms selectively consume diatoms. These complex behaviors of macroinvertebrates make them a critical group to consider when developing appropriate toxicity assays to establish regulatory limits for contaminants.

A shortcoming of the EPA-developed criteria is that the toxicity bioassays used to generate toxicity data do not consider dietary exposures. Failure to account for the trophic transfer of dietary contaminants is a limitation of such tests in the context of real ecosystem processes. Metal toxicity studies for aquatic macroinvertebrates have shown that diet is important and can be a predominant exposure pathway for contaminants [17,18,19,20]. Organisms that graze on biofilms can accumulate metals from the environment and show the greatest disconnect between lab and field data [11]. However, since regulatory bodies assume that dietary toxicity is less important than aqueous exposure to free metal ions based on the argument that the surface action of metals (rather than bioaccumulation) better predicts acute toxicity and is seldom used in developing regulatory frameworks, this kind of data is rarely generated [11,16]. Nonetheless, the available data show that the dietary toxicity pathway can be toxic to grazers for metals [20,21]. Thus, the dietary contribution to toxicity needs to be assessed to set a protective regulatory limit.

The chemical speciation models used to predict bioavailable copper assume a conventional organic matter type while modeling NOM regardless of the source of the organic matter. However, copper complexation with NOM can vary in strength depending on the source of the NOM [22]. Luan and Vadas [23] found that effluent organic matter had a higher binding capacity for metals than stormwater, even with a lower organic matter concentration. This implies that at the same organic matter concentration, effluent organic matter would translate into less bioavailable metals for both the organisms and their diets. Regulatory limits set from experiments conducted with effluent organic matter and metal speciation modeled with generic NOM might fail to prove effective in protecting organisms when they are exposed to episodic metal loading from stormwater runoffs.

This study aims to determine the toxicity of copper to the freshwater amphipod *H. azteca* under ecologically relevant exposure conditions. We investigate the effect of pre-exposing the diet and the experimental system to the exposure water. We also compare two diet types, including the more commonly used flake food and the most likely available diet to organisms in their natural habitat, periphyton. Finally, we also investigate the effect of two different sources of exposure water with different organic matter types. Our results indicate a more toxic effect under exposure conditions pre-equilibrated to the exposure water and with natural dietary sources and less toxic effects in the presence of effluent organic matter compared with stormwater organic matter.

## 2. Materials and Methods

This study was designed to investigate the lethal and sublethal effects of 14 d dietary Cu exposures on *H. azteca* with varying water types and food sources.

### 2.1. Treatment Water Setup for Exposure Conditions

The exposure water was made with natural water sources, either collected stormwater flow within 24 h of a major storm event in a suburban stream (Hockanum River in Vernon, CT, USA) or wastewater treatment plant effluent (Storrs, CT, USA). Organic matter concentrations were adjusted by dilution and set at 2.5 and 4 mg L^−1^ as representative concentrations in urban streams. Stormwater exposure waters were adjusted with 10% humic acid (Suwannee River Humic Acid Standard II) in addition to dilution. Exposure water backgrounds were adjusted to moderately hard reconstituted water (MHWR) [24] with NaHCO_3_, CaSO_4_·2H_2_O, MgSO_4_, and KCl as necessary. The pH of the water samples was adjusted to 7.5 with NaOH or H_2_SO_4_. Five metal concentrations were selected in 15 μg L^−1^ increments with the lowest concentration being 50 μg L^−1^ in order to envelop both the lethal and sublethal effects of copper over the range of exposure conditions.

### 2.2. Treatment of Diet

Periphyton colonization was adapted from published procedures [25]. Approximately 100 frosted microscope slides (76 × 25 mm) were preloaded into glass microscope slide racks and suspended in 38 L colonization aquariums for three to five weeks to allow for mature biofilm communities to develop. At the start of colonization, periphyton inoculum was introduced into the aquariums by adding a rock collected from the Fenton River, Connecticut. The aquariums were filled with approximately 23 L of water from the Fenton River and exchanged on a weekly basis. The river water has low background copper concentrations (about 1.0 µg/L) and no major wastewater or stormwater inputs leading to low nutrient concentrations. The aquariums were spiked with nutrients every other day with approximately 15 µg/L phosphorus (sodium phosphate dibasic) and 0.2 mg/L nitrogen (ammonium chloride and sodium nitrate each) to encourage faster growth. Each aquarium contained two submersible pumps to circulate water and contained an aeration system to supply sufficient dissolved oxygen and carbonate concentrations. Illumination was provided by fluorescent lights situated perpendicular to the growing surfaces of the frosted slides. The photoperiod was 14:10 h light–dark, and the average light irradiance was 300 µmol m^−2^ s^−1^. TetraMin^®^ Tropical Flakes, manufactured by Tetra^®^, were used as a commercial diet and are referred to as TetraMin^®^ throughout this paper. When pre-equilibration of the diet was used, the diet was equilibrated with the corresponding exposure waters for 48 h prior to the start of the experiment. This period was determined experimentally to be enough time for the periphyton to come to equilibrium under these conditions.

### 2.3. Exposure Setup

Acid-washed (10% HNO_3_) 600-mL polypropylene beakers were used as exposure vessels for all the experiments. All vessels were pre-equilibrated for 24 h with the corresponding exposure water. Plastic transfer pipettes were used to transfer 5 organisms to each beaker. All experiments had 3 replicates. The water-to-diet ratio was kept considerably higher in order to keep the nominal concentration of the exposure water within acceptable limits. The diet was introduced to the exposure setup on day 0 for the unequilibrated experiments. For the pre-equilibrated experiments, the beakers were equilibrated with the exposure water along with the diet. Organisms were introduced after the equilibration period. Water samples (5 mL) were analyzed for metals and major cations from each container after each water replacement.

### 2.4. Toxicity Bioassay

Three-day-old organisms were obtained from the Columbia Environmental Research Center (CERC), Columbia, MO, USA, and were acclimated in the laboratory from days 4 to 6. The experiments commenced with 7-day-old organisms.

The exposure beakers were covered with plastic film to reduce evaporation loss. Loss was measured by weighing a random representative beaker regularly during the course of the experiment. The test was a 14-day non-renewal test with no aeration. The test temperature was 23 ± 1 °C. We used photoperiod 12 h ambient laboratory lighting with about 500 lux. The toxicity bioassay parameters are tabulated in Table S1.

### 2.5. Post-Exposure Treatment

After the experiment beakers were sampled for final copper concentration (5 mL), dissolved oxygen (DO) and pH were checked. The organisms were collected with disposable pipettes. They were rinsed. The organisms were counted for survival. All organisms in each beaker were photographed (iPhone 15 pro) along with a ruler for reference, and the images were analyzed with ImageJ software (version 1.54g) [26].

### 2.6. Analytical Chemistry

Total dissolved organic carbon concentrations were measured using a Shimadzu TOC-V (Shimadzu, Columbia, MD, USA). Calibration and quality control (QC) standards were prepared from two different potassium hydrogen phthalate sources (Ricca Chemical, Arlington, TX, USA; Fisher Scientific, Waltham, MA, USA). The instrument and method blanks were included in all sample analysis batches; QC samples were run periodically, which met the expected concentrations within 20%.

Concentrations of Cu were measured on an Agilent 7700 inductively coupled plasma mass spectrometer (ICP-MS; Santa Clara, CA, USA) with a helium collision cell. Samples were acidified to 1% using 70% trace metal grade HNO_3_ before analysis. Calibration and QC standards were prepared from independent high-purity sources (VHG Labs, Manchester, NH, USA; SpexCertiprep, Metuchen, NJ, USA). Scandium was used as an internal standard, and all QC standards were within 10% or better of expected values.

### 2.7. Toxicity Data Analysis

The survival percentage for the replicate beakers was calculated, and survival in control beakers for each experiment was checked against an acceptable limit. Percent survivals were used to calculate 50% lethal concentration, LC50. The organisms’ lengths were analyzed with ANOVA for beaker effects. In the case of a beaker effect, a beaker-averaged length for each replicate beaker was used to calculate 20% effect (reduction in length) concentrations, i.e., EC20 values.

All models were fit using the R statistical computing platform with the associated .drc package [27]. There are several models in the package. Initially, every model was ranked using the Akaike information criterion (AIC). The models were then assessed in no particular order according to the following: residual variance, the lack of fit test, and the number of model parameters compared to the number of observations.

A subset of models that performed the best with respect to these initial metrics were then fit and assessed. The primary metric of concern was significance tests on each of the model parameters. A model was rejected unless all parameters were deemed significant. The LC_50_/EC_20_ estimates were examined, and standard errors relative to the point estimates were compared. Following that, a subjective visual examination of the fitted model and 95% confidence bands of the model was generated.

## 3. Results

This study reports results on the aqueous concentrations and resultant dose–response plots of LC50 and EC20 (length) based on the previously described experimental controls and various treatments. The control experiments satisfied the acceptability criteria (lowest survival 86.7%) for survival (>80%) and exposure water conditions during the test [24]. The context of each is focused on either more carefully controlled speciation in the system or providing more realistic conditions for the organisms. Also in this context, the noted nominal concentrations specify the target Cu concentration for the experiment, whereas the measured Cu concentrations represent the final measured concentrations in the experimental solution. We first compare the sources of organic matter, including stormwater and effluent organic matter, and then consider the influence of diet that is either pre-equilibrated with solution or not.

### 3.1. Control of Experiments

**Figure 1:**
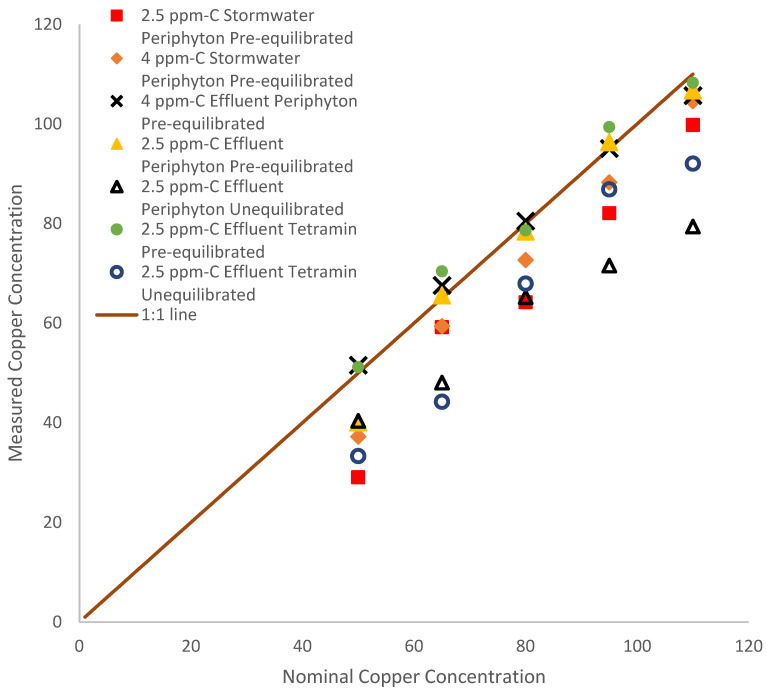
Measured vs. nominal copper after the 14-day exposure experiment. Squares, diamonds, crosses, triangles, pluses, filled circles, and hollow circles represent 2.5 ppm stormwater, 4 ppm, stormwater, 4 ppm effluent, 2.5 ppm effluent periphyton pre-equilibrated, 2.5 ppm effluent periphyton unequilibrated, 2.5 ppm effluent TetraMin^®^ pre-equilibrated, and 2.5 ppm effluent TetraMin^®^ unequilibrated, respectively. The brown line represents the 1:1 line.

In terms of aqueous concentrations of Cu, the measured versus nominal concentrations of Cu in the experimental solutions were compared. The nominal Cu concentrations were nearly identical to the measured Cu concentrations only in the case of effluent organic matter or systems that had pre-equilibrated dietary sources with effluent organic matter (Figure 1). In all other cases, with either stormwater organic matter or unequilibrated diets, the nominal concentrations were higher than measured values, meaning that losses of Cu from solution occurred. Some evaporation losses were observed over the course of the 14-day experiment, but only about 0.1% per day, which would normally result in more concentrated solutions and does not account for some of the larger drops in concentration that were observed. In the case of stormwater versus effluent organic matter where the measured values dropped by as much as 43%, the losses were likely driven by the lower ligand concentrations and binding constants in the stormwater, allowing for more exchange to various surfaces in the system compared with systems with effluent organic matter [28]. In the case of different dietary sources, the lower measured Cu concentrations in the unequilibrated diets by as much as 26% were very likely due to the redistribution of aqueous Cu to the diet over the course of the experiment, leading to varied aqueous and dietary exposures. The unequilibrated TetraMin^®^ experiment showed the largest discrepancy between the measured and nominal concentrations.

### 3.2. Effect of Pre-Treatment of Diet

Equilibration of the exposure media to the experimental system is important to obtain a clear measure of the aqueous Cu concentration. It is also important in the context of natural systems because of the interaction with dietary sources of exposure. We examined both lethal and sublethal (organism length) effects in toxicity assays that were fed the commercial TetraMin^®^ diet versus the periphyton natural food source in scenarios where they were either added at the start of the experiment or pre-equilibrated with the exposure media.

**Figure 2:**
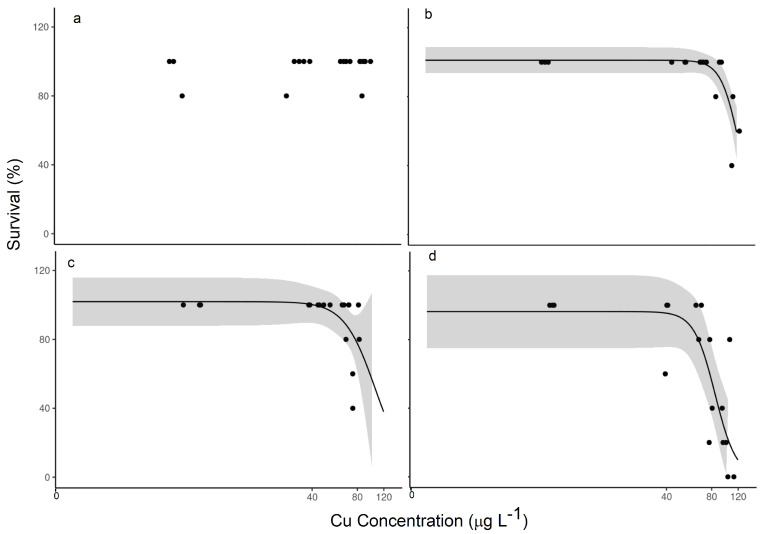
Relationships between measured copper concentration (μg L^−1^) and % survival following 14-day exposures: (**a**) effluent TetraMin^®^ unequilibrated; (**b**) effluent TetraMin^®^ pre-equilibrated; (**c**) effluent periphyton unequilibrated; and (**d**) effluent periphyton pre-equilibrated. The shaded region represents the confidence band. All exposures contained 2.5 ppm organic carbon.

In the traditional assay, with the addition of unequilibrated TetraMin^®^ as the food supply, over the range of 4.5 (background) to 99.5 µg/L Cu, we observed very little impact on *H. azteca* with only three random deaths out of 75 organisms across the range and an indeterminate value of LC50 (Figure 2a). In the experiment with unequilibrated periphyton, we started to observe lethality at 65 µg/L Cu (Figure 2c) and calculated an LC50 value of 105.6 µg/L Cu (Table 1). That stark difference alone is a concern, but when we equilibrated the food supply with the exposure media, as it would be in the natural environment, further differences were observed. The LC50 value for *H. azteca* fed with pre-equilibrated TetraMin^®^ showed an effect this time and was 125.7 µg/L Cu, and it was even lower in the case of periphyton, at 84.3 µg/L Cu.

**Figure 3:**
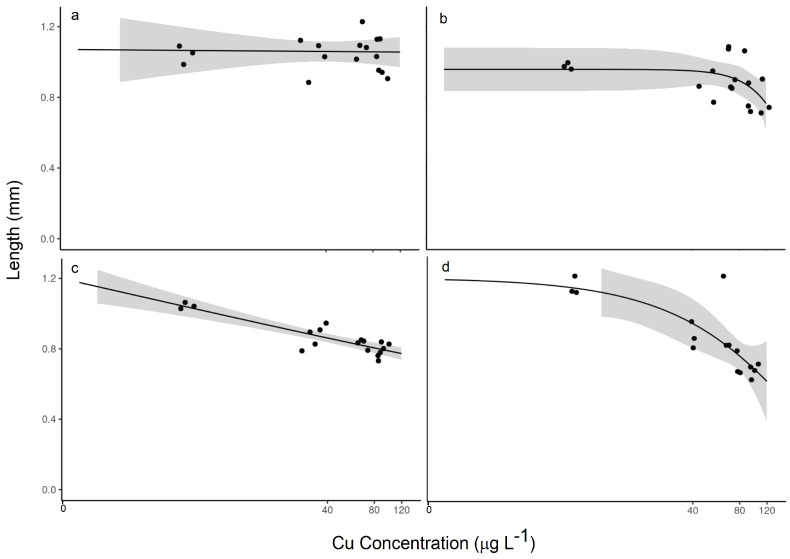
Relationships between measured copper concentration (μg L^−1^) and length (mm) following 14-day exposures: (**a**) effluent TetraMin^®^ unequilibrated; (**b**) effluent TetraMin^®^ pre-equilibrated; (**c**) effluent periphyton unequilibrated; and (**d**) effluent periphyton pre-equilibrated. The shaded region represents the confidence band. All exposures contained 2.5 ppm organic carbon.

The difference in impact was even more stark in the measured effect on length (Figure 3). Again, the unequilibrated TetraMin^®^-fed organisms showed no distinct response, and the EC20 value was indeterminate over this range of Cu exposure. Yet, the organisms exposed to unequilibrated periphyton showed a distinct decrease in length from about 1.06 mm at the lowest exposure concentration to about 0.73 mm at the highest exposure concentration, but the EC20 value was indeterminate. In experiments with pre-equilibrated food supplies, decreased lengths were observed in both cases again, with a greater impact observed in the *H. azteca* exposed to pre-equilibrated periphyton, with an EC20 value of 39.6 µg/L Cu, which was 67% lower compared with the EC20 value of 119.3 µg/L Cu in the organisms exposed to pre-equilibrated TetraMin^®^.

When we consider nominal exposure media Cu concentrations, meaning the concentration of Cu that we targeted to establish the consistent aqueous concentration, compared with the measured Cu concentrations after equilibration with all aspects of the exposure system (primarily container and diet), even greater differences were observed in the calculated LC50 or EC20 values (Figures S1–S4). For example, Table 1 shows that considering nominal concentrations would not quantify a 50% lethality concentration or a 20% effect concentration for the pre-equilibrated traditional lab diet. Moreover, for the pre-equilibrated natural diet, the measured concentrations show a 20% lower EC20 value. In terms of sublethal effects, neither diet showed any valid dose–response in the unequilibrated diet exposure experiments, whereas the pre-equilibrated exposures showed an EC20 for periphyton that was at least 54% lower than that of TetraMin^®^. This means equilibration is important not only for the correct exposures in the experiments but also for quantifying the toxicity parameters correctly. This effect will be most important when dietary exposure is considered.

### 3.3. Effect of the Organic Matter Source

Another aspect of natural systems that is not always considered is the variation in organic matter sources. In particular, in more urban systems, we distinguished stormwater sources and wastewater effluent sources and again measured the influence on lethal and sublethal (length) toxicity effects in the experiments. In this case, all experiments used pre-equilibrated periphyton as the dietary source.

**Figure 4:**
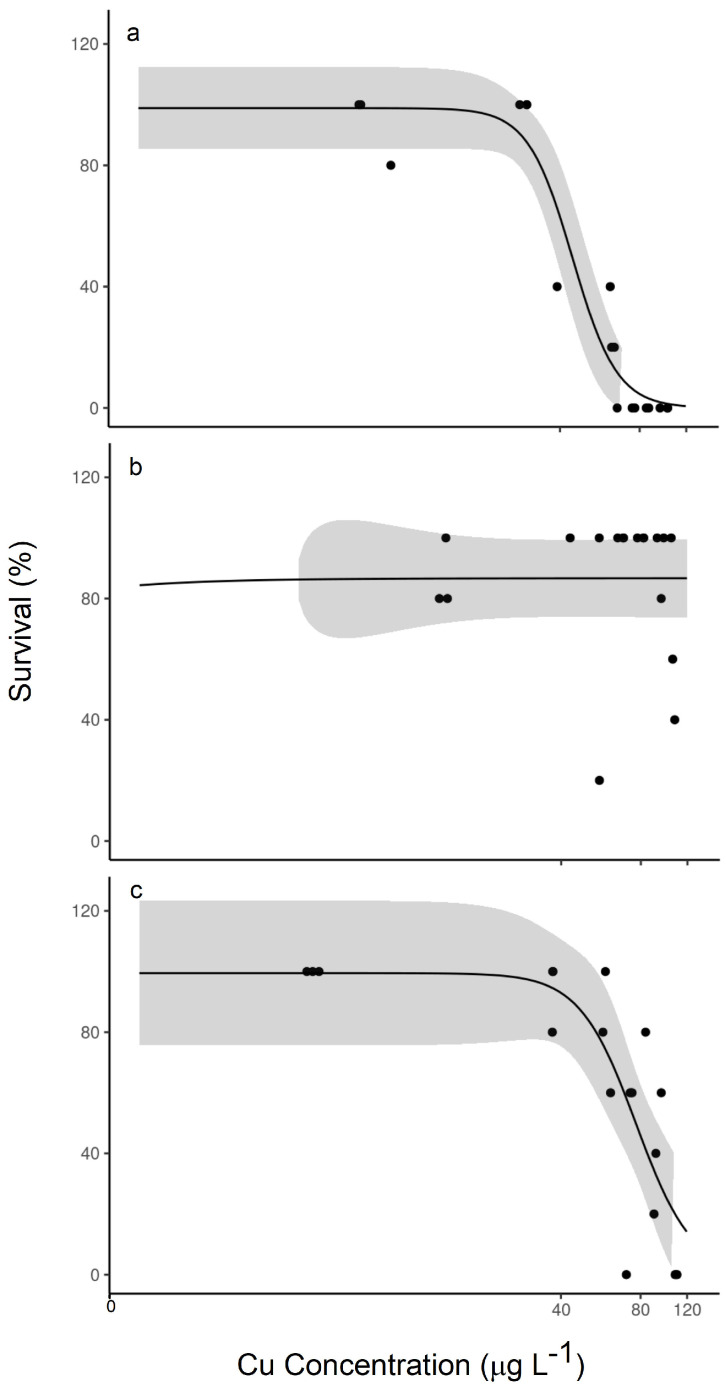
Relationships between measured copper concentration (μg L^−1^) and % survival following 14-day exposures: (**a**) stormwater periphyton pre-equilibrated; (**b**) effluent periphyton pre-equilibrated; and (**c**) stormwater periphyton pre-equilibrated. The shaded region represents the confidence band. (**a**) contained 2.5 ppm organic carbon and (**b**,**c**) contained 4 ppm organic carbon.

Organic matter source had a clear influence on lethality over the same range of exposure concentrations. In comparison with the LC50 value of 84.3 μg/L Cu observed above (Table 1) with 2.5 ppm effluent and pre-equilibrated periphyton as the diet, the same experiment, but with stormwater organic matter, resulted in an LC50 value of almost less than half, at 44.6 μg/L Cu (Figure 4a). As the concentration of organic matter was slightly increased in the experiment to 4 ppm C, we observed no distinct effect with effluent exposure (Figure 4b). Lethality did sometimes occur at the higher exposure concentrations, but the LC50 values were indeterminate (likely greater than 110 μg/L). Lethality was observed in the 4 ppm stormwater C exposure, with a lower LC50 value of 76.9 μg/L Cu (Figure 4c). In these observations, the source of organic matter clearly influences lethality, with effluent organic matter being more protective, and a higher concentration of organic matter leading to lower lethality rates in both sources.

**Figure 5:**
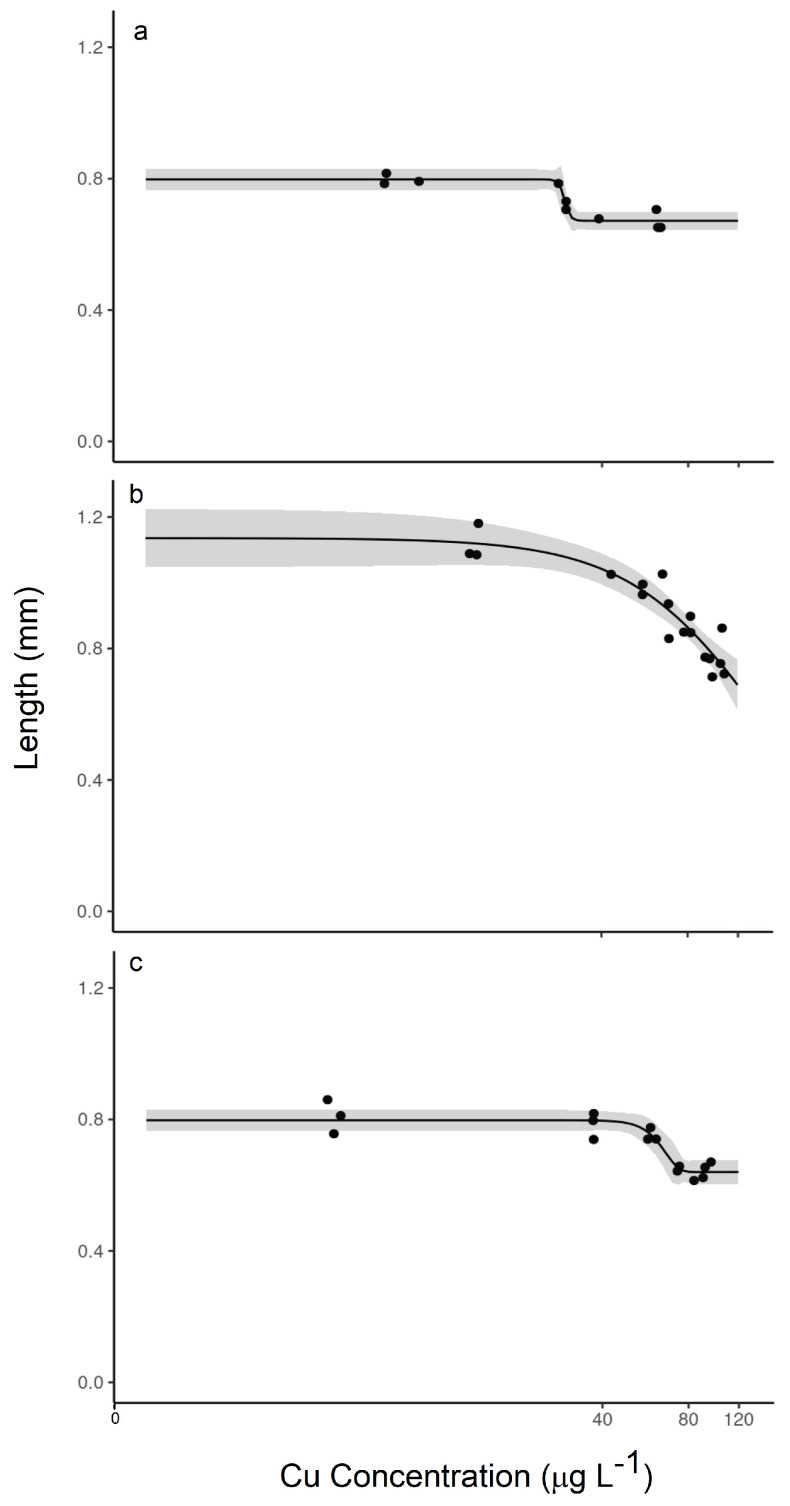
Relationships between measured copper concentration (μg L^−1^) and length (mm) following 14-day exposures: (**a**) stormwater periphyton pre-equilibrated; (**b**) effluent periphyton pre-equilibrated; and (**c**) stormwater periphyton pre-equilibrated. The shaded region represents the confidence band. (**a**) contained 2.5 ppm organic carbon and (**b**,**c**) contained 4 ppm organic carbon.

The differences in non-lethal effects on *H. azteca* when exposed to different organic matter sources mirrored the lethal effects. Compared with the EC20 value of 39.6 μg/L Cu in the 2.5 ppm effluent C exposure (Figure 3d), the EC20 value in the 2.5 ppm stormwater C exposure was almost 28% lower at 28.7 μg/L Cu (Figure 5a). Those differences became smaller as the C concentration increased to 4 ppm, with EC20 values of 56.7 μg/L Cu for the effluent, which was only about 20% lower than the 70.5 μg/L Cu for the stormwater (Figure 5b,c).

## 4. Discussion

The results of this study show the significant influence of some experimental conditions when using laboratory toxicity bioassays to set regulatory limits for copper. Some of the assumptions or simplifications used while conducting toxicity assays, especially when considering dietary exposures and subsequently developing numeric criteria can affect the practical applicability of the established regulatory limits. Recognizing that the nominal metal concentration is not always the actual metal concentration can lead to higher-than-expected LC50 values, translating to higher numeric criteria. In addition, carefully equilibrating the experimental system with both the apparatus and dietary supply can lead to larger differences in the apparent LC50 and EC20 values. Our results show that toxicity assays conducted in the laboratory with the unequilibrated diet can underestimate lethal toxicity in assays performed with the natural diet of the organism, periphyton. In the case of the more commonly used diet in these kinds of experiments, i.e., the flake food TetraMin^®^, the unequilibrated diet showed no effect, whereas the pre-equilibrated diet showed a much higher percent lethality at a relatively higher concentration. Furthermore, the source of OM altered the partitioning and uptake of Cu to the diet and ultimately to *H. azteca*, resulting in lower LC50 and substantially lower EC20 values in the systems with stormwater versus effluent OM. Each of these changes in how a toxicity assay is run can ultimately affect the established numeric criteria for Cu in surface waters and even regulatory WET results.

Currently, the standard assay for *H. azteca* that is used to establish Cu surface water criteria is an acute assay with no specific attention to the dietary source [24]. The two current *H. azteca* datasets for Cu exposure that the U.S. EPA uses with the BLM to establish surface water criteria [29] are 96 h toxicity bioassays designed to quantify acute toxicity. The organisms used were a range of ages but within an age group known to consume a significant amount of diet. The experiments were conducted with a flake food diet provided to the system. The concern with dietary exposure, especially in such short-term assays, is that the diet can adsorb Cu from the solution, thus decreasing the dissolved concentration of Cu in the exposure water and effectively changing the exposure condition in real time. This loss of metals from the exposure water is a known issue in toxicity assays, and some studies have reactively measured and reported the final concentration in the exposure water [30,31] as one way to address this discrepancy. However, that represents a dynamic exposure scenario. Particularly for *H. azteca*, Borgman et al. [32] found that Cu accumulated over one week (body burden increased 2.5-fold), but after 10 weeks, the body burden was not significantly different from the control as Cu uptake slowed down and any accumulated Cu was diluted by growth. Any changing concentration in a toxicity assay would result in a variable response from the organism as it adapts to regulate the metal or as the metal exerts additional stress on the organism. This discrepancy in Cu exposure during a toxicity assay can stem from a few factors, including the equilibration of the exposure media with the apparatus or with the dietary source. These factors could be exacerbated depending on the makeup of the exposure water, with the organic matter source playing a particularly important role for Cu.

The equilibration of the exposure media with the apparatus and dietary sources can have a large impact on the reported toxic effects or the LC50 and EC20 values. With the unequilibrated systems, we observed up to about a 40% drop in the measured concentration as Cu partitioned either to the container or the dietary source, meaning that the aqueous exposure of Cu was lower than we anticipated. In these cases, we could not always determine a specific LC50 or EC20 value, but if we compare the dose–response curves, for example, in a setup with effluent OM and unequilibrated periphyton as the diet, we can see a clear shift in the effects to a higher concentration in the nominal (Figure S1) versus the measured (Figure 3c) exposure concentrations, which would shift the EC20 value to a higher concentration. The equilibration of the system, with both the apparatus and the diet, resulted in reported LC50 values that were nearly the same between the nominal and measured concentrations in the case of the effluent OM with pre-equilibrated periphyton (Table S1), although the EC20 values were still about 20% different.

Even within systems that are pre-equilibrated, the source of organic matter becomes more important. The systems with stormwater as the OM source had larger differences in the nominal versus measured Cu concentrations compared with effluent OM, which translated to larger differences in the reported LC50 and EC20 values. For example, whereas the effluent OM with pre-equilibrated periphyton had nearly the same LC50 values in the nominal versus measured plots, the stormwater LC50 value shifted 35% higher in the nominal plot, and the EC20 value almost doubled. Bigger shifts like this can have a large influence on the numeric criteria developed for surface water quality, suggesting it is very important to know and maintain the exposure concentration more precisely in order to determine the toxic effect of certain water quality. Pre-treating food in the exposure water in order to bring the food to equilibrium with the exposure conditions is one way to achieve this [16]. Static or intermediate renewal of overlaying water, or flowthrough systems, can also address this issue to some degree. However, static renewal can impose a shock load on the organisms and still effectively create a dynamic exposure condition. On the other hand, intermediate renewal and flowthrough systems require large volumes of exposure water and come with the risk of losing diet from the system.

Beyond the shift in exposure concentrations, the dietary source plays a big role in toxic effects. Laboratory-based assays commonly feed commercial diets such as TetraMin^®^ or similar flake food and yeast–Cerophyll(R) trout chow (YCT). However, in our system, we fed periphyton, a mixture of bacteria and algae that grows on streambed surfaces and is one of the natural food sources for grazers in stream ecosystems. *H. azteca* fed with periphyton, whether pre-equilibrated or not, always showed toxic effects, both lethal and non-lethal, at lower concentrations than TetraMin^®^-fed organisms. In fact, in the unequilibrated systems, TetraMin^®^-fed organisms showed no effect across the range of Cu exposure concentrations. TetraMin^®^ adsorbed similar amounts of Cu from solution as periphyton at the lower concentrations of less than 80 µg/L, suggesting the aqueous exposure was the same and the differences were primarily due to diet. As Cu concentrations increased, periphyton adsorbed more, by almost double, resulting in higher dietary concentrations that could have driven the observations of lethality starting at those concentrations. However, non-lethal effects were also observed at lower concentrations in the unequilibrated periphyton that were not observed with TetraMin^®^, suggesting that the food source itself and not just the dietary Cu concentration influenced the toxicity.

The contribution of diet to toxicity becomes more important when using periphyton as the diet [33], which has greater implications for chronic toxicity assays. The addition of diet nearly always reduces the aqueous exposure concentrations if not pre-equilibrated, but this could differ depending on the diet used in terms of both equilibrium dietary concentrations as well as the rate of uptake. In this case, while the partitioning of Cu to TetraMin^®^ is primarily a physical–chemical process driven by adsorption, the natural periphyton diet also actively takes up Cu and often can accumulate Cu to much higher concentrations, as we observed here [33,34]. However, that biological uptake process is slower than mere adsorption, as we found periphyton needed at least 48 h to come to equilibrium. For laboratory-based acute exposure scenarios, the role of diet could play an outsized role depending on the diet, as both the timing and partitioning of Cu from the aqueous to the dietary phase resulted in very different exposure conditions over a short 96 hr exposure, whereas, in chronic exposure scenarios, diet is likely the dominant source of copper to the organism. While chronic toxicity assays with *H. azteca* inherently need a diet, our results show that even in systems where the diet is pre-equilibrated with the exposure media and both aqueous and dietary exposures are more tightly controlled, the periphyton-fed organisms had substantially lower LC50 and EC20 values, by about 35% and 65%, respectively. This shows the importance of designing experiments that represent environmentally relevant conditions.

In each case, whether aqueous or dietary exposure, OM plays a role in controlling the bioavailability of metals, as anticipated and used in the BLM-based establishment of water quality criteria for Cu [29]; however, the source of OM is also important to consider. The BLM model currently uses WHAM II [35] to establish Cu speciation, in which up to 60% of natural organic matter (NOM) can be considered inert in terms of binding copper [36]. However, this is misleading as that model was developed with purely natural organic matter in mind, assuming a fraction of either fulvic acids (FAs) or humic acids (HAs). However, the organic matter present in urban systems has some distinct differences [28] that can change bioavailability and ultimately toxicity. Cu exposure with effluent OM is expected to have lower bioavailability compared with stormwater OM owing to higher metal–OM complexation. However, in a system with both aqueous and dietary exposure, the interplay between OM and both the apparatus and the diet needs to be considered.

The results show that the source of organic matter has significant effects in terms of both lethal and sublethal toxicity. Stormwater was shown to have a more toxic effect in comparison with effluent as the source of organic matter. In both cases, with 2.5 ppm and 4 ppm carbon concentrations, lethal and sublethal effects were higher in the case of stormwater organic matter, and the differences in effects were higher at the lower organic matter concentration. This was expected because a higher carbon concentration increases copper complexation and thus reduces bioavailability. Higher lethal and sublethal effects of stormwater can be explained by the binding ligand characteristics of OM sources. For example, [28] showed that while stormwater, from the same source used here, had a higher (0.163 μM mg^−1^) concentration of binding ligands than that of an effluent source (0.119 μM mg^−1^), the effluent source had a stronger ligand (20% hydrophobic with a conditional stability constant, log K of 8.4–9.3 and 80% hydrophilic with a log K of 8.0–8.4) compared with the effluent (77% hydrophobic with a log K of 7.9–8.0 and 23% hydrophilic with a log K of 7.2–7.8). While the activity of free copper is accepted as more important to bioavailability [37], ligand-bound copper (Cu-L) [38] could also contribute to copper bioavailability and the accumulation pathway [39]. Copper weakly bound to ligands can also dissociate from the complex because of its strong affinity to biotic ligands or other conditions near an organism’s surface [28]. This implies that at lower concentrations, the effect can be magnified even further since the concentration of strong binding ligands in stormwater will be minimal, rendering more of the copper bioavailable to organisms than in the presence of effluent organic matter. Furthermore, copper can be taken up by an organism via the dietborne pathway [11] and the diet can accumulate more copper from stormwater as well [28].

While research has attributed copper toxicity to the effects of ligands and cations on copper bioavailability, these are not the only controlling factors. For example, acute copper toxicity in aquatic organisms has been related to the disruption of osmoregulation, specifically sodium/potassium exchange [40,41,42,43], which can be affected by calcium rather than by competition with copper for the same biochemical receptor. Similarly, the reported effects of sodium and potassium on copper toxicity [44] might simply reflect favorable or unfavorable ion exchange gradients, rather than any effect on copper bioavailability. Nevertheless, the effects of ligand complexation and cation competition on copper bioavailability provide a reasonable conceptual framework for improved descriptions of how copper toxicity differs across exposure conditions.

Regulatory control is one of the practical implications of the results of this study that shows the impacts of the test parameters on the test results. Under the Clean Water Act, the EPA established the National Pollutant Discharge Elimination System (NPDES) program in order to regulate individual direct dischargers of pollutants, both publicly owned treatment works (POTWs) and industrial facilities, as well as polluted stormwater dischargers. Under this program, the EPA uses whole effluent toxicity (WET) tests to identify effluents and receiving waters containing toxic materials in chronically toxic concentrations. The data from these tests are used for NPDES permit development and to determine compliance with permit toxicity limits. These data can also be used to predict potential acute and chronic toxicity in the receiving water. The tests are performed as a part of self-monitoring permit requirements, compliance biomonitoring inspections, toxic sampling inspections, and special investigations. Our results imply that discharges that are impacted by stormwater can significantly alter the bioavailability of the associated copper present. Hence, if a WET test is performed using synthetic dilution water, the results can significantly underestimate the actual toxicity of the whole effluent.

Although chronic toxicity data show both lethal and sublethal effects of the BLM parameters used in the EPA BLM-Cu criteria, they do not set the freshwater chronic limit the same way as the acute limit because of insufficient data. The chronic criterion was calculated by multiplying an acute-to-chronic ratio (ACR) [15] to the FAV at different conditions to derive a Final Chronic Value (FCV), for which there are several assumptions and limitations. One of the assumptions is the ACR reasonably approximates the bioavailability relationships for chronic toxicity from the FAV. This criterion uses two macroinvertebrates, i.e., *G. pseudolimnaeus* and *H. azteca*, to calculate the FAV. Only one of them, *G. pseudolimnaeus*, falls under the fifth percentile of toxicity values of the GMAV. However, the criterion does not use any of these species to calculate the ACR. The EPA has a standard sediment toxicity bioassay for both acute and 10-day chronic tests for *H. azteca* but no aqueous toxicity bioassay. Furthermore, in the 10-day chronic tests, organisms are fed but dietary toxicity is not specifically considered. Historically, studies use similar bioassays for water-only tests. The GMAV for the genus *H. azteca* at the reference condition for the EPA Cu-BLM criteria was 12.07 μgL^−1^. A 20% reduction in this value (9.65 μgL^−1)^ takes it closer to the GMAV for another amphipod genus, Gammarus (9.60 μgL^−1^), which is within the fifth percentile of the GMAVs. This implies that *H. azteca* might not be protected to the degree intended by the regulatory limit set by the current criteria.

## 5. Conclusions

This study found that, in terms of short-term chronic copper toxicity for the freshwater crustacean *H. azteca*, some of the important aspects of the experimental setup are the pre-equilibration of the exposure setup and the diet before the exposure. This has significance in developing ecologically grounded toxicity assays, which can be used to develop refined WQCs, especially for metals. This aspect becomes more important in assays with natural living diets for macroinvertebrates than commercial non-living diets. The natural diet also showed higher lethal and non-lethal toxicity for copper to *H. azteca*, which has implications for standardized dietary toxicity bioassays used both for setting regulatory pollutant limits and for evaluating compliance with those regulations. The organic matter source in exposure water also plays an important role in the toxicity of *H. azteca*. Copper exposure via stormwater showed higher toxic effects for both the lethal and non-lethal endpoints. This has significance in setting site-specific WQCs for metals in different sites impaired with pollutants from different sources. This also has significance in compliance evaluation sampling schedules regarding these pollutant limits. This study points out some possible improvements in the short-term chronic toxicity assays for *H. azteca*. This research can be expanded to investigate other toxicity endpoints such as full lifecycle survival and length as well as reproductivity. Another avenue to investigate could be the maternal transfer of copper and its effects on the next generation of *H. azteca*. Potential future research directions include studying the effects of similar parameters (i.e., pre-equilibration, use of natural diets, and organic matter sources) for other toxic pollutants that have the potential of partitioning with the experimental setup, accumulating in the diets of aquatic organisms, and rendering altered bioavailability in presence of organic matter for organisms for which the dietary exposure pathway can be significant.

## Figures and Tables

**Table 1:** LC50 and EC20 values calculated in μg/L based on either nominal or measured exposure concentrations for different diets and organic matter sources.

OM Source	Diet	Equilibration	Organic Carbon Conc.	Based on Nominal Cu (µg/L)		Based on Measured Cu (µg/L)	
			mg/L	LC50	EC20	LC50	EC20
Effluent	Commercial diet	Unequilibrated	2.5	Id	Id	Id	Id
		Pre-equilibrated	2.5	Id	Id	125.7	119.3
Effluent	Natural diet	Unequilibrated	2.5	Id	183.5	105.6	Id
		Pre-equilibrated	2.5	83.2	50.4	84.3	39.6
Stormwater	Natural diet	Pre-equilibrated	2.5	60.3	53.5	44.6	28.7
Effluent	Natural diet	Pre-equilibrated	4	Id	Id	>105.7	70.5
Stormwater	Natural diet	Pre-equilibrated	4	82.0	61.1	76.9	56.7

Id = indeterminant effects concentration. Commercial diet = TetraMin^®^. Natural diet = periphyton.

## Data Availability

The raw data supporting the conclusions of this article will be made available by the authors on request.

## Supplementary Materials

The following supporting information can be downloaded at https://www.mdpi.com/article/10.3390/toxics12080608/s1, Table S1: Toxicity bioassay parameters; Figure S1: Relationships between nominal copper concentration (μg L^−1^) and length (mm) following 14-day exposures: (**a**) effluent TetraMin^®^ unequilibrated; (**b**) effluent TetraMin^®^ pre-equilibrated; (**c**) effluent periphyton unequilibrated; and (**d**) effluent periphyton pre-equilibrated. The shaded region represents the confidence band; Figure S2: Relationships between nominal copper concentration (μg L^−1^) and length (mm) following 14-day exposures: (**a**) effluent TetraMin^®^ unequilibrated; (**b**) effluent TetraMin^®^ pre-equilibrated; (**c**) effluent periphyton unequilibrated; and (**d**) effluent periphyton pre-equilibrated. The shaded region represents the confidence band; Figure S3: Relationships between nominal copper concentration (μg L^−1^) and % survival following 14-day exposures: (**a**) stormwater periphyton pre-equilibrated; (**b**) effluent periphyton pre-equilibrated; and (**c**) stormwater periphyton pre-equilibrated. The shaded region represents the confidence band; Figure S4: Relationships between nominal copper concentration (μg L^−1^) and length (mm) following 14-day exposures: (**a**) stormwater periphyton pre-equilibrated; (**b**) effluent periphyton pre-equilibrated; and (**c**) stormwater periphyton pre-equilibrated. The shaded region represents the confidence band.
