# Supplementary material for: Short-Term Chronic Toxicity of Copper to Hyalella azteca: Contrast in Terms of Equilibrating Diet, Diet Type, and Organic Matter Source
Source: Toxics. 2024 Aug 20;12(8):608. doi: 10.3390/toxics12080608 (PMC11358939; doi:10.3390/toxics12080608)
Supplement: Supplementary file 1 [file toxics-12-00608-s001.zip › toxics-3136253-supplementary.pdf]

Short-Term Chronic Toxicity of Copper to Hyalella azteca: Contrast in Terms of Equilibrating Diet, Diet Type, and Organic Matter Source

Nafis Fuad [1,*], Timothy Vadas [1] and Rebecca Williams [2]

[1] Department of Civil and Environmental Engineering, College of Engineering, University of Connecticut, Storrs-Mansfield, CT 06269, USA; nafis.fuad@uconn.edu
[2] Department of Civil, Construction and Environmental Engineering, College of Engineering, North Carolina State University, Raleigh, NC 27695, USA; rlwill26@ncsu.edu
* Correspondence: nafis.fuad@uconn.edu; Tel.: +1-860-3086949

Supplementary Data

Table S1: Toxicity bioassay parameters.

Figure S1: Relationships between nominal copper concentration (μg L^{-1}) and % survival following 14-day exposures: (a) Effluent Tetramin® untreated; (b) Effluent Tetramin® pretreated; (c) Effluent Periphyton untreated; (d) Effluent Periphyton pretreated. The shaded region represents confidence band. All exposures contained 2.5 ppm organic carbon.

Figure S2: Relationships between nominal copper concentration (μg L-1) and length (mm) following 14-day exposures: (a) Effluent Tetramin® untreated; (b) Effluent Tetramin® pretreated; (c) Effluent Periphyton untreated; (d) Effluent Periphyton pretreated. The shaded region represents confidence band. All exposures contained 2.5 ppm organic carbon.

Figure S3: Relationships between nominal copper concentration (μg L-1) and % survival following 14-day exposures: (a) Stormwater Periphyton pretreated; (b) Effluent Periphyton pretreated; (c) Stormwater Periphyton pretreated. The shaded region represents confidence band. (a) contained 2.5 ppm organic carbon, (b) & (c) contained 4 ppm organic carbon.

Figure S4: Relationships between nominal copper concentration (μg L-1) and length (mm) following 14-day exposures: (a) Stormwater Periphyton pretreated; (b) Effluent Periphyton pretreated; (c) Stormwater Periphyton pretreated. The shaded region represents confidence band. (a) contained 2.5 ppm organic carbon, (b) & (c) contained 4 ppm organic carbon.

Table S1: Toxicity bioassay parameters

**Test parameter**	**Value**
Test species	*Hyalella azteca*
Test type	Non-renewal
Test Duration	14 days
Temperature	23±1
Light quality	Ambient laboratory light
Light intensity	500 lux
Photoperiod	12L:12D
Test chamber size	600-mL high-form beaker
Test chamber material	Polypropylene
Renewal of solution	None
Test solution volume	400 mL
Age of test organism	7-d known-age, acclimated in laboratory from day 4-6
Number of organisms per test chamber	5
Number of replicate chambers per concentration	3
Feeding	Pretreated or untreated Periphyton colonized slides or Tetramin® flakes on day 0
Aeration	None
Overlaying water	Collected Stormwater and Wastewater effluent adjusted to properties of MHRW and corresponding dissolved organic carbon
Overlaying water quality	Dissolved oxygen measured at 14 days
Nominal exposure Cu concentrations	Control, 50, 65, 80, 95, 110
Endpoint	Day 14 survival and length
Test acceptability criterion	80% survival in control exposure

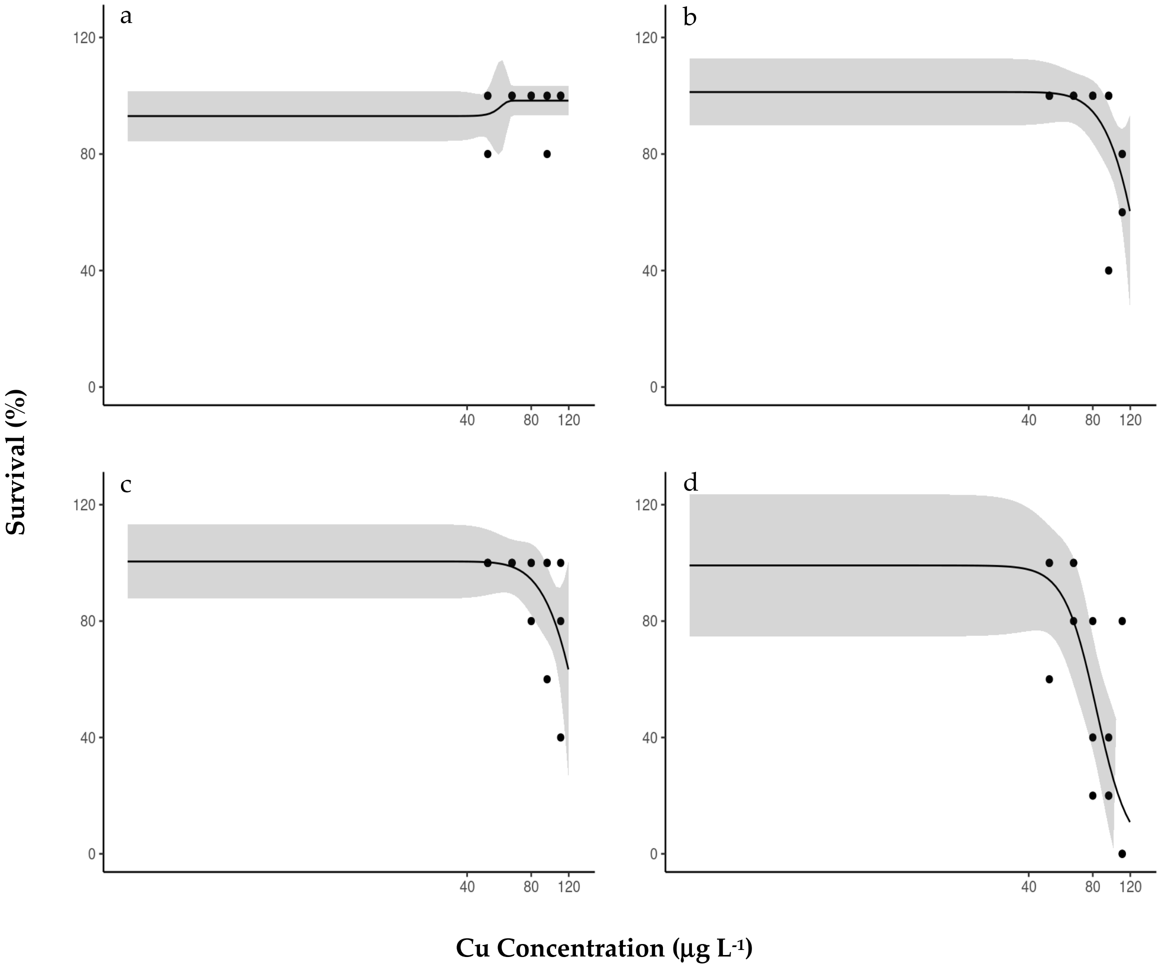

Figure S1 Relationships between nominal copper concentration (µg L^{-1}) and % survival following 14-day exposures: (**a**) Effluent Tetramin® untreated; (**b**) Effluent Tetramin® pretreated; (**c**) Effluent Periphyton untreated; (**d**) Effluent Periphyton pretreated. The shaded region represents confidence band. All exposures contained 2.5 ppm organic carbon.

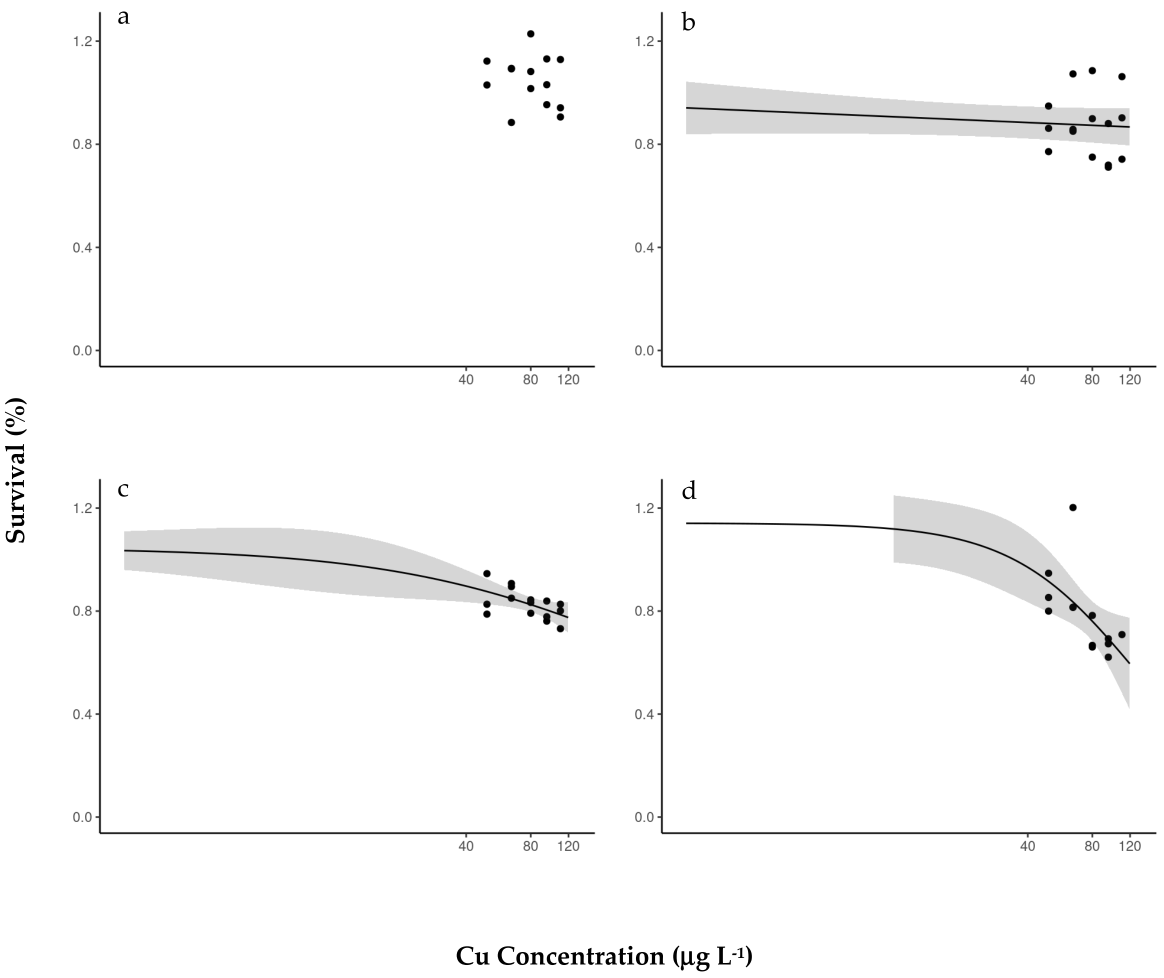

Figure S2 Relationships between nominal copper concentration (μg L^{-1}) and length (mm) following 14-day exposures: (**a**) Effluent Tetramin® untreated; (**b**) Effluent Tetramin® pretreated; (**c**) Effluent Periphyton untreated; (**d**) Effluent Periphyton pretreated. The shaded region represents confidence band. All exposures contained 2.5 ppm organic carbon.

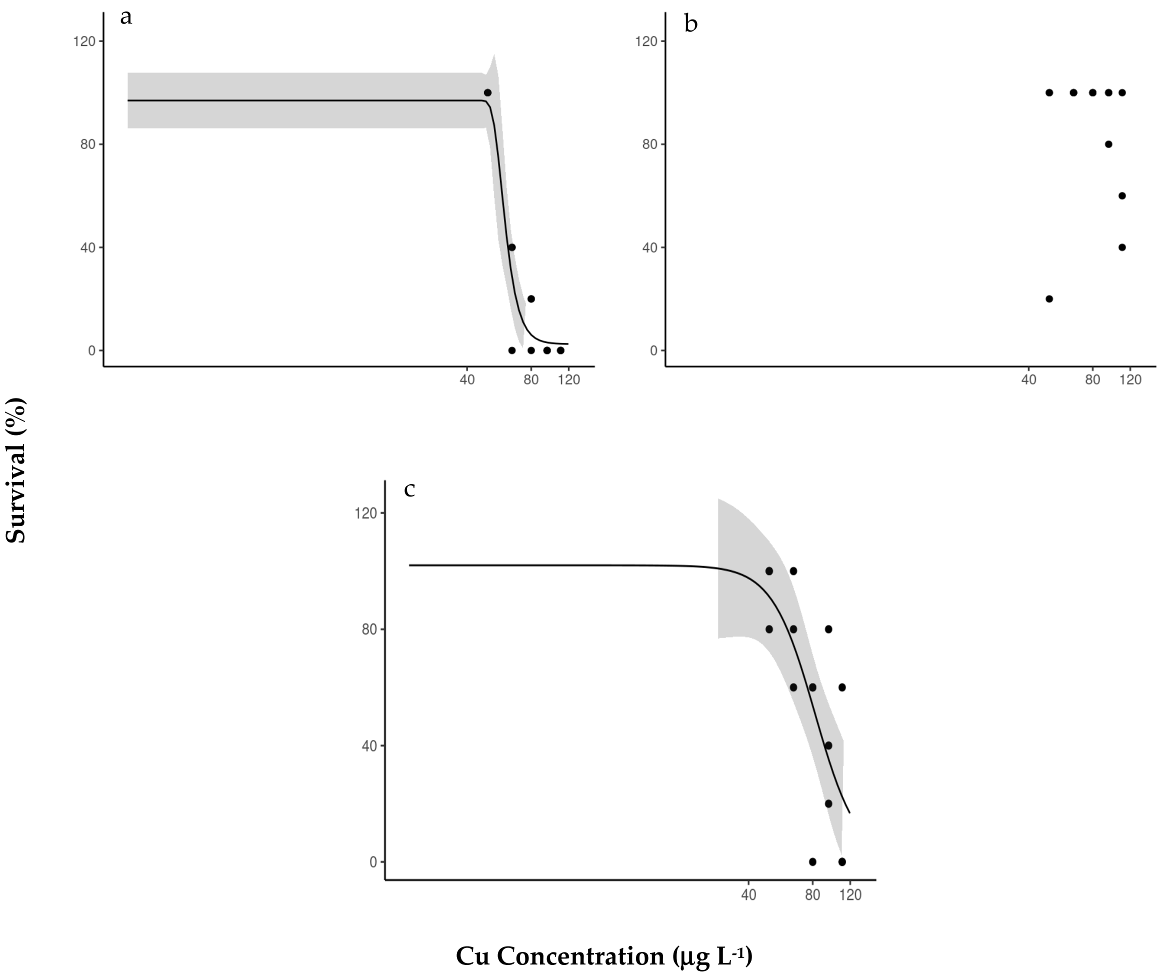

Figure S3 Relationships between nominal copper concentration (µg L^{-1}) and % survival following 14-day exposures: (**a**) Stormwater Periphyton pretreated; (**b**) Effluent Periphyton pretreated; (**c**) Stormwater Periphyton pretreated. The shaded region represents confidence band. (a) contained 2.5 ppm organic carbon, (b) & (c) contained 4 ppm organic carbon.

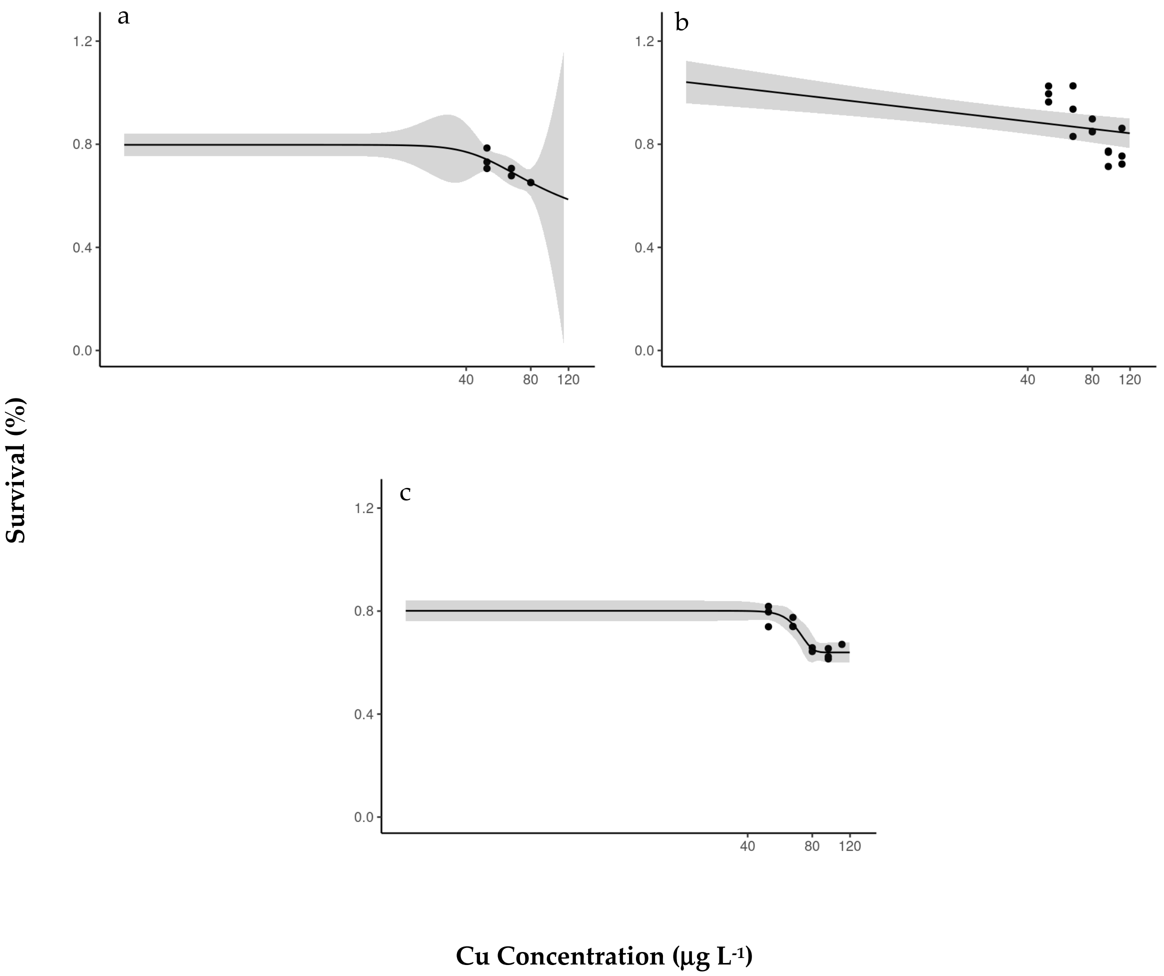

Figure S4 Relationships between nominal copper concentration (µg L^{-1}) and length (mm) following 14-day exposures: (**a**) Stormwater Periphyton pretreated; (**b**) Effluent Periphyton pretreated; (**c**) Stormwater Periphyton pretreated. The shaded region represents confidence band. (a) contained 2.5 ppm organic carbon, (b) & (c) contained 4 ppm organic carbon.
